# An Atomic Force Microscopy analysis of Exosomes derived from Tumor Associated pluripotent Mesenchymal Stem Cells

**DOI:** 10.26502/fjhs.388

**Published:** 2026-01-09

**Authors:** Tanmay Kulkarni, Simone Newell, Erik Armstrong, Narendra Banerjee, Jasmine Cuffee, Hirendra Banerjee, Santanu Bhattacharya

**Affiliations:** 1 Department of Biochemistry and Molecular Biology, Mayo Clinic College of Medicine and Science, 4500 San Pablo Road South, Jacksonville, FL, 32224, USA; 2 Department of Natural, Pharmacy and Health Sciences, Elizabeth City State University campus of The University of North Carolina, Elizabeth City, NC 27909

**Keywords:** Exosomes, Mesenchymal Stem Cells, Atomic Force Microscopy, Cancer Biomarker

## Abstract

Mesenchymal stem cell (MSC)-derived exosomes are recognized as critical mediators within the tumor microenvironment (TME), exerting both pro- and anti-tumorigenic effects depending on contextual factors. These vesicles are also gaining attention for their potential as therapeutic vehicles in regenerative medicine and targeted drug delivery. However, the influence of the TME on the physical characteristics of MSC-derived exosomes remains poorly understood. In this study, we utilized Atomic Force Microscopy (AFM) to investigate the morphological and nanomechanical properties of MSC-derived exosomes under standard and TME-like conditions. AFM imaging in fluid mode preserved the native structure of exosomes and enabled high-resolution analysis of their topography, surface roughness, stiffness, adhesion, and deformation. AFM offers unique advantages in exosome research by enabling label-free, nanoscale analysis of vesicle properties in near-physiological conditions. The ability to detect such subtle but functionally significant changes highlights the relevance of AFM in exosome characterization and quality assessment. Our results revealed that exposure to the TME induces marked changes in exosomal membrane morphology and mechanical behavior, including increased surface heterogeneity, higher stiffness, and altered adhesive interactions. These biophysical alterations may reflect changes in membrane composition and protein or lipid cargo, potentially affecting exosome function, uptake, and therapeutic efficacy. Overall, our findings provide new insights into how the TME modulates MSC exosome biophysics and underscore the utility of AFM-based techniques for advancing the development of exosome-based diagnostics and therapies.

## Introduction

The tumor microenvironment (TME) is a complex and dynamic niche composed of a heterogeneous population of cells, signaling molecules, vasculature, and the extracellular matrix (ECM) [1, 2]. Among the non-malignant cells present in the TME, mesenchymal stem cells (MSCs) play a pivotal role in modulating tumor progression[3]. A key mechanism through which MSCs influence the TME is via the secretion of exosomes, nano-scale vesicles loaded with bio-molecular cargo such as RNA, DNA, and proteins[4, 5]. These exosomes function as critical messengers, orchestrating a variety of cellular processes that promote tumor growth and metastasis[6, 7]. MSCs are multipotent stromal cells that can differentiate into various cell types, including osteoblasts, chondrocytes, and adipocytes[8]. In the context of cancer, they are integral components of the TME, contributing to the formation of tumor stroma and facilitating the anchorage of tumor cells[9]. Moreover, MSCs can transdifferentiate into other cell types such as M2-type macrophages and myeloid-derived suppressor cells (MDSCs), which further enhance the pro-tumorigenic milieu[10, 11].

Exosomes have emerged as a promising platform for drug delivery due to their unique properties and functional characteristics[12, 13]. These nanosized extracellular vesicles, typically ranging from 30 to 150 nm in diameter, demonstrate significant advantages over traditional drug delivery systems such as liposomes and synthetic nanoparticles [14]. Their innate features include low toxicity, high biocompatibility, and the ability to cross biological barriers, including the crucial blood-brain barrier (BBB)[15, 16]. Among the various sources of exosomes, those secreted by mesenchymal stem cells are gaining increasing attention for their roles in modulating the TME [5, 17]. These vesicles are capable of delivering diverse molecular cargo directly into recipient cells, thereby influencing key cellular pathways and promoting tumor progression. Emerging evidence suggests that these exosomes play a significant role in the suppression of anti-tumor immunity and the development of resistance to cancer therapies [18, 19]. This indicates their pathological involvement in tumor growth and progression. Atomic Force Microscopy (AFM) is a versatile and powerful tool that has found widespread application in biological research due to its capacity for high-resolution imaging, nanomechanical characterization, and operation in near-physiological environments [20]. Unlike electron microscopy, which often requires extensive sample preparation, AFM enables label-free, real-time imaging of biological samples under native conditions, preserving structural integrity and functionality [21]. In cellular and molecular biology, AFM has been extensively employed to probe the surface topography and mechanical properties of various biomaterials, including cells, membranes, proteins, and exosomes [22-29]. Its ability to perform force spectroscopy allows precise quantification of nanomechanical parameters such as stiffness (Young’s modulus), adhesion, and deformation, which are critical indicators of physiological or pathological changes at the nanoscale [30]. AFM enables the visualization of exosomal morphology with nanometer resolution, distinguishing subtle alterations that might be undetectable by techniques like nanoparticle tracking analysis (NTA) or dynamic light scattering (DLS) and transmission electron microscopy (TEM). Furthermore, AFM-based nanomechanical profiling can reveal changes in exosome stiffness and elasticity, which are associated with disease progression, including cancer [31, 32].

**Figure 1:**
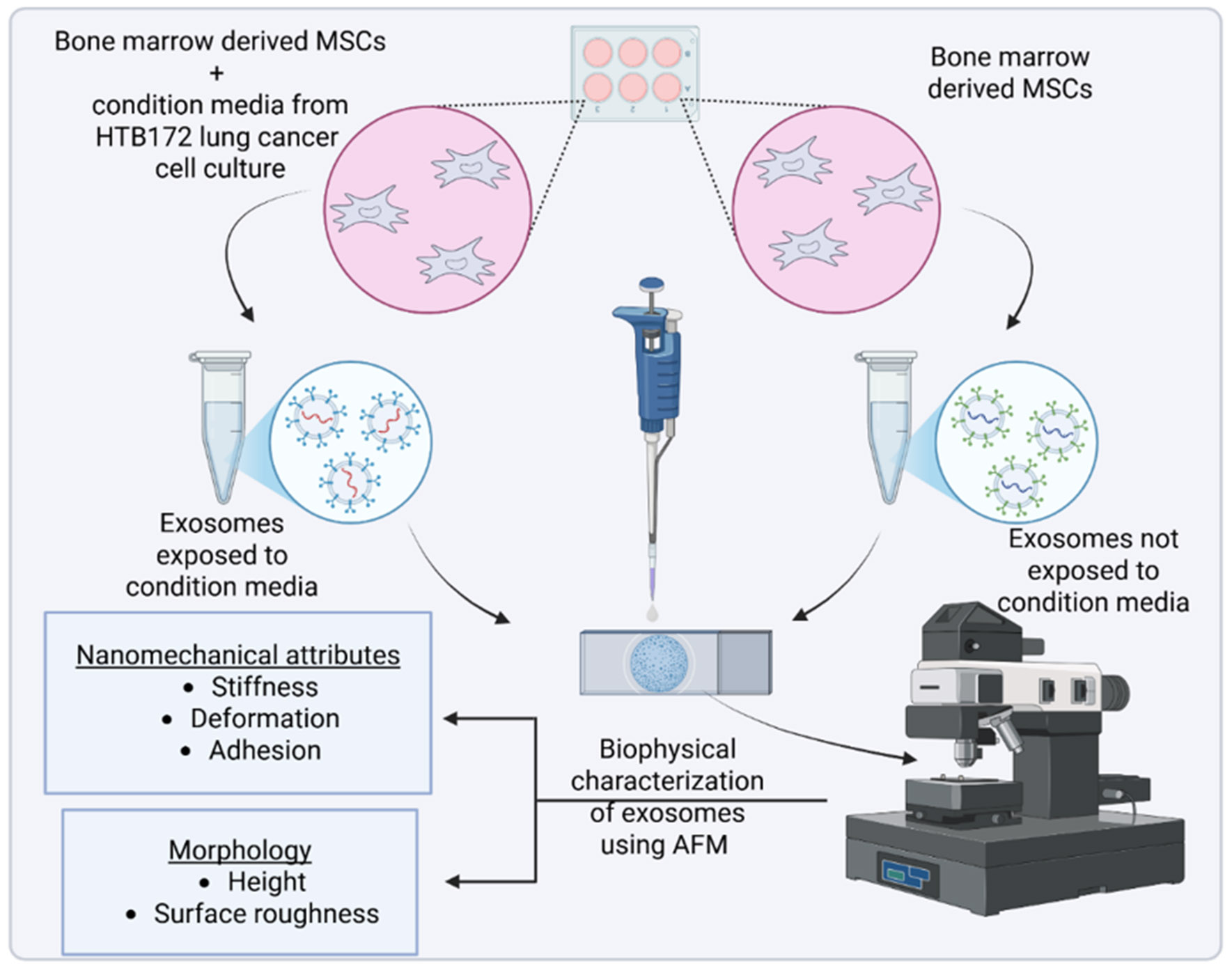
A schematic illustration of experimental methodology from exosomes isolation to their nanomechanical characterization.

Recent studies have demonstrated that tumor-derived or tumor-exposed exosomes exhibit distinct mechanical properties compared to those from healthy or unexposed cells. These nanomechanical signatures, measurable by AFM, may serve as potential biomarkers for diagnosis or therapeutic targeting [33] . The combination of AFM imaging and force spectroscopy thus provides a comprehensive biophysical characterization of exosomes, facilitating a deeper understanding of their roles in the tumor microenvironment (TME) and their impact on cellular behavior. In this study, we utilized AFM to investigate the morphological and nanomechanical differences between exosomes derived from Mesenchymal Stem Cells (MSCs) exposed to condition media from lung culture and those from unexposed MSCs. An experimental schematic overlaying the process is shown in Figure 1. This approach aims to elucidate how the TME influences exosomal biophysical properties including morphology and mechanical behavior.

## Materials and Methods

### Cell Culture

NCl-H209 (ATCC HTB-172), Bone marrow-derived mesenchymal stem cells (ATCC PCS-500-12) were acquired from American Type Culture Collection (ATCC, Manassas, Virginia). Routine maintenance for each cell line was followed as per ATCC protocol. All media (ATCC PCS-500-030) were supplemented with 10% fetal bovine serum and 100 μg/mL penicillin/streptomycin. All cell lines were grown in 5% CO_2_ in 25 cm^2^ filter cap flasks and cultured in a laboratory incubator at 37°C.

### MSC-TME Exposure

In six-well plates, MSC cells were grown to confluence, placed under inserts with 0.4 μm micropores, which contained HTB172 lung cancer cells grown to confluence along with controls with no cancer cells. These cells were cultured for a period of 48 hours before exosomes were isolated.

### Exosome Isolation

Exosomes were isolated following the total exosome isolation kit purchased from Invitrogen, Carlsbad, CA (Catalog # 4478359). As per the manufacturer’s protocol, from a confluent flask of cells, culture media was collected in a 15 mL centrifuge tube and centrifuged at 14,000 rpm, 10 min. Supernatant was discarded and the pellet was re-suspended in 5 mL of appropriate incomplete media (no Fetal Bovine Serum), placed in a 25 cm^2^ culture flask, and allowed to grow for up to 12 h. This was done to eliminate the high levels of exosomes contained in FBS and to prevent contamination of the cell derived exosomes. After growing cells in incomplete media for 10 h, cells in media were collected into a 15 mL centrifuge tube and centrifuged at 14,000 rpm for 10 min. Supernatant (1 mL) was placed in each of 5 Eppendorf tubes (1.5 mL). Supernatant was then centrifuged at 2,000 x g for 30 min to remove cells and debris and supernatant was then transferred from each tube and placed into a new 1.5 mL Eppendorf tube without disturbing the pellet (total 5 tubes). Total Exosome Isolation reagent (0.5 volumes) was added to each of the 5 tubes containing cell-free culture media. The culture media/reagent mixture was vortexed until a homogenous solution appeared, and the mixture was incubated overnight at 4°C. After incubation, the samples were centrifuged at 10,000 x g for 1 hour at 4°C in the benchtop Centrifuge 5804 R (Eppendorf, Hauppauge, NY). The supernatant was aspirated and discarded leaving the pellet containing exosomes, which was not readily visible in most cases. The pellet was then resuspended in 1 mL 1X PBS and stored in minus eighty-degree Celsius freezer. Exosomes were validated by CD 63 Elisa kit with appropriate control.

### AFM experimental methodology

Freshly cleaved mica surface, which is optimally flat, possesses a slight negative charge is commonly used to study nm-sized biological samples [34, 35]. Herein, it was employed to study the morphology and nanomechanical attributes of exosomes under various treatments. To achieve an effective adsorption of exosomes on mica surface, freshly cleaved mica was modified using a 3:1 mixture by volume comprising of 3-aminopropyltriethoxysilane (APTES) and N, N-diisopropylethylamine (DIPEA) for 2 hours at 60 °C [15]. The modified mica surface was used immediately. The salinization process makes the mica surface to be positively charged enabling effective adsorption of negatively charged exosomes. For AFM experiments, the exosome samples were diluted 1000-fold with Milli Q water. Herein, we used Milli Q water instead of PBS (pH 7.4) to avoid salt formation that may affect morphological and nanomechanical scans. Working exosome sample was prepared by diluting the original exosome stock solution 1000-fold with Milli Q water to avoid salt deposition from PBS. Following which, 5 μL of working solution was drop-casted on the modified mica to let the exosomes adhere for 30 mins. Mica surface was then rinsed with Milli Q water to remove any unbound exosomes. To conduct AFM experiments, we employed Dimension Icon Scanasyst AFM (Bruker Corporation, Santa Barbara, CA). To evaluate the surface morphology and nanomechanical attributes of the exosomes, we incorporated Peak Force Quantitative Nanomechanical Mapping (PF-QNM) and Nanoindentation experimental techniques, respectively. All the experiments were performed in liquid environments to preserve exosome morphology and nanomechanical attributes. PFQNM, a specialized tapping mode generates a height image and quantitates nanomechanical attribute of the sample simultaneously. In Peak force tapping mode, where the AFM tip intermittently meets the sample surface to generate a feedback signal yielding a surface topography. A Scanasyst Air probe bearing a sharp tip has a radius of 5 nm and a stiff cantilever with nominal spring constant of 0.4 N/m and with pyramidal tip geometry was used to achieve high resolution topography of the exosomes. For characterization of topographical morphology, the laser was aligned on the back of the cantilever (gold reflective surface) to achieve an optimal signal-to-noise ratio. Topography was assessed using a low peak force of 300 pN and scan rate of 0.1 Hz. At least 50 samples were analyzed to evaluate the surface topography of exosomes. Nanoscope Analysis v1.9 software was then employed to analyze morphological characteristics such as the height and surface roughness of the exosomes. The height of the exosomes was analyzed using the sectional analysis module under the analysis software whereas, the surface roughness was derived as the root mean square variation in the feature height, also available as an inbuilt module. Surface roughness was calculated by precisely selecting the region over the exosomes. Care was taken such that the selected region does not incorporate any background. For nanomechanical properties characterization, AFM probe was calibrated in the presence of Milli Q water on a freshly cleaved mica surface to determine the actual spring constant of 0.38 N/m and deflection sensitivity of 35 nm/V. The calibration of AFM tip in fluid medium allows compensation for the hydrodynamic drag that exists during the AFM experiments. Nanoindentation experiment was performed on at least 50 samples in which each tip-sample interaction resulted into a force-separation (F-S) curve. A trigger force of 600 pN was applied to engage the tip with the sample. Further, by employing DMT model, each F-S curve was analyzed to yield various nanomechanical attributes such as Young’s modulus (YM), deformation and adhesion. DMT model was employed as the indentation was comparable to overall sample dimensions (few tens of nm) as well as distinct adhesion force that was observed during tip-sample interaction [36].

### Principal Component Analysis

To reveal the exposure effect of condition media from HTB172 lung cancer cell culture on MSC-derived exosomes, we performed Principal Component Analysis (PCA). We utilized the raw nanomechanical attribute data from stiffness, deformation and adhesion both from exposed and non-exposed treatments. Principal component analysis (PCA) was performed to explore multivariate differences between non-exposed (n = 50) and exposed (n = 50) independent samples. Prior to PCA, data were standardized by mean-centering and scaling to unit variance. PCA was conducted using a covariance-based approach, and the first two principal components (PC1 and PC2), which together account for 100% of the total variance, were retained for visualization. PCA scores were plotted as PC1 versus PC2 using GraphPad Prism, with each point representing an individual sample and samples colored according to exposure status. Distribution of samples across all four quadrants reflects natural biological variability around the dataset mean and does not imply directional bias. Group-level patterns were assessed based on relative separation and centroid position along the principal components.

### Data Analysis

To analyze the data from the AFM, we utilized Nanoscope analysis v1.9 software proprietary from Bruker. The height was determined by using sectioning tool displaying the 3-dimenional topography of the exosomes. Each F-S curve was baseline corrected before applying DMT model. We evaluated at least 50 exosome samples for each treatment. Furthermore, GraphPad Prism was used to plot the graphs and One-Way ANNOVA was used as the choice of statistical significance. Before employing One-Way ANNOVA, the dataset was evaluated for normality test.

## Results

### Morphology of MSC-Derived Exosomes Under Various Conditions

**Figure 2:**
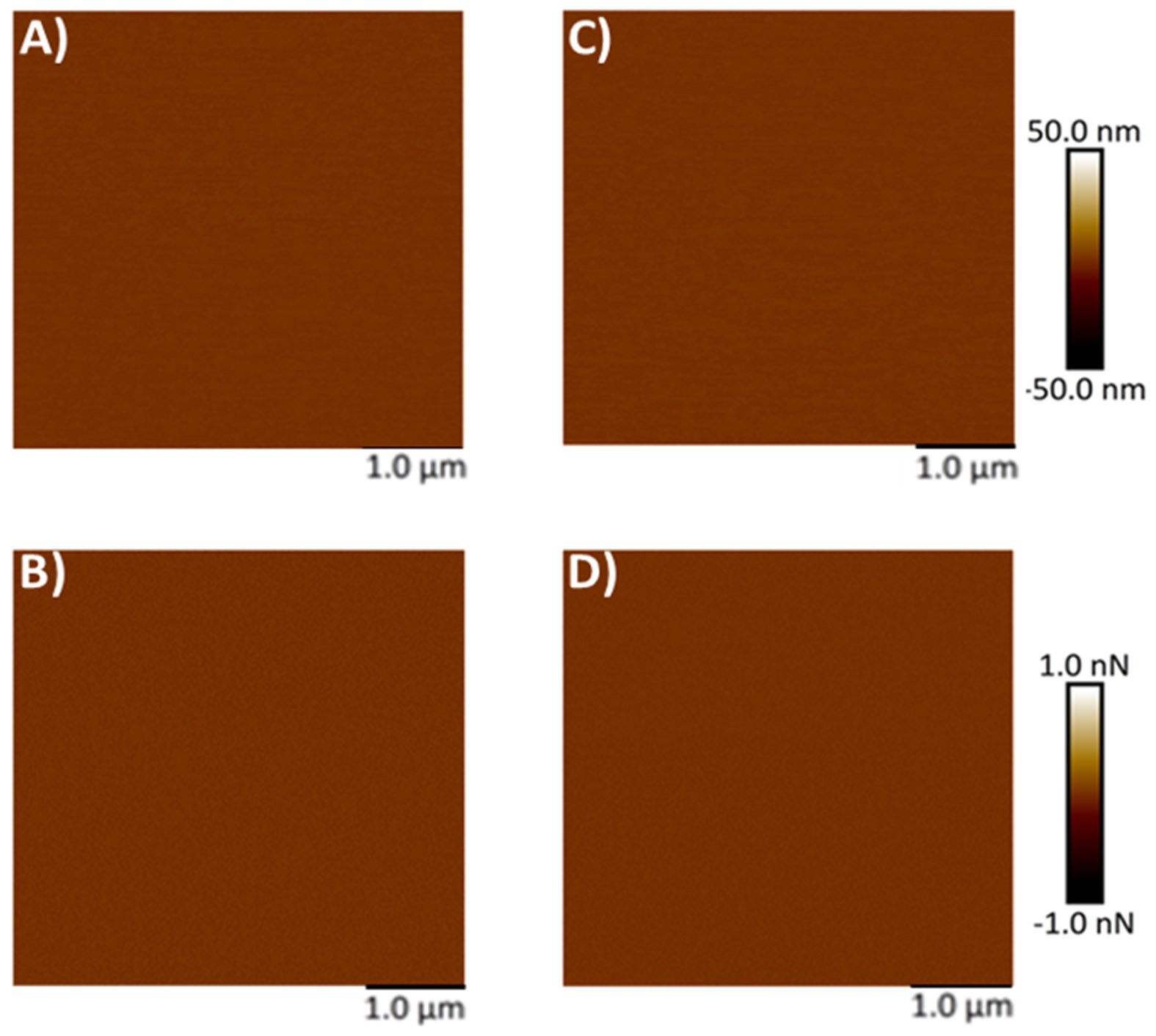
Representative surface topography of mica surface subjected to modifications. A) Height image and B) Peak force error image of **freshly cleaved mica surface.** C) Height image and D) Peak force error image of **APTES modified mica surface.**

**Figure 3:**
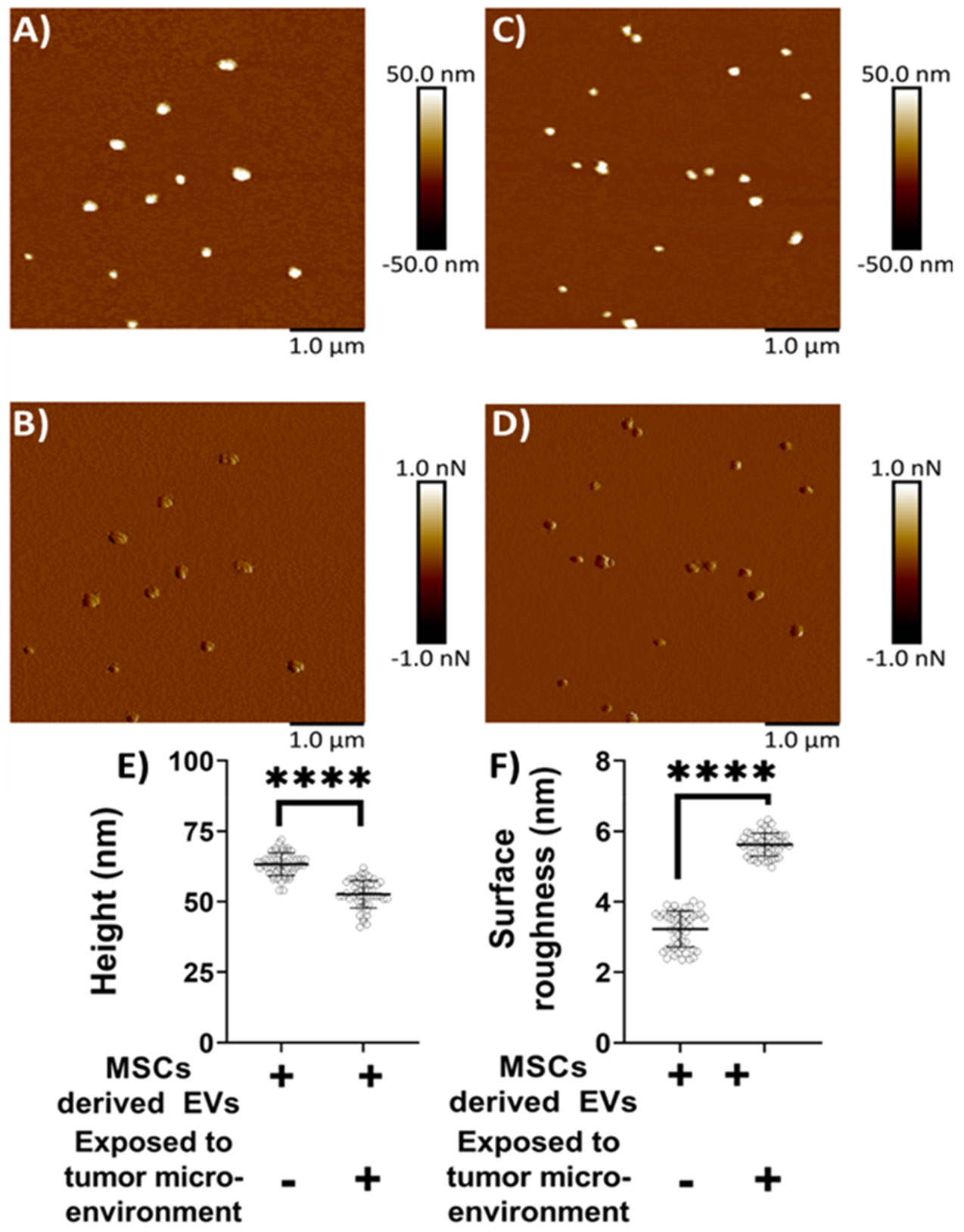
Representative surface topography of mesenchymal stem cells (MSC) derived exosomes under various conditions. A) Height image and B) Peak force error image of MSC derived exosomes non-exposed to TME. C) Height image and D) Peak force error image of MSC derived exosomes exposed to TME. Morphology quantifications. E) Height. F) Surface roughness. (Statistical significance performed by One Way ANNOVA: ****, p<0.0001)

Mica, being optimally flat with a slightly negative surface charge, is commonly preferred for AFM studies [26-28, 34, 35]. To facilitate the adsorption of negatively charged exosomes, the mica surface was rendered positively charged by treatment with APTES: DIPEA. Initially, AFM was used to assess the topography of both freshly cleaved mica and mica following APTES: DIPEA treatment. Representative height and peak force error images for freshly cleaved mica surface and APTES treated surface are shown in Figures 2A-2B and 2C-2D, respectively. No significant changes in surface topography were observed post-treatment. The surface roughness of freshly cleaved mica and salinized mica was observed to be 0.465 nm and 0.583 nm, respectively. Further, Figures 3A-3B represent height image and peak force error image, respectively, of exosomes derived from MSCs. Although the peak force error image provides qualitative insights, quantitative morphological analysis was performed on the corresponding height images. The peak force error image reflects the feedback signal captured during PFQNM mode. Figures 3C-3D display height image and peak force error image, respectively, of exosomes derived from MSCs exposed to conditioned media from lung cancer cell culture (TME like environment). To assess potential morphological differences, height images from both TME-exposed and non-exposed MSC-derived exosomes were analyzed to quantify average surface roughness and height profiles. Morphology features exhibiting a size less than 100 nm were chosen, those justifying exosomes. We also observed particle size greater than 100 nm, which we neglected as they could be group of exosomes stacking to higher particle size. These experiments were repeated independently on three occasions, and additional representative data are included in Supplementary Figure S1. AFM morphology imaging was conducted using probes with sharp tips, minimizing the contact area and enabling detailed resolution of surface features, as supported by previous literature [37, 38]. A reduced interaction area allows more precise detection of sudden topographical variations, yielding high-definition morphological data [25]. The topographical maps are obtained in a raster scanning format. Exosomes derived from MSCs not exposed to the TME were found to be significantly larger in height (63.28 ± 4.05 nm) compared to those from TME-exposed MSCs (52.66 ± 4.90 nm), as shown in Figure 3E. Conversely, MSC-derived exosomes not exposed to the TME exhibited significantly lower topographical variation (3.22 ± 0.51 nm) relative to TME-exposed exosomes (5.62 ± 0.32 nm), as illustrated in Figure 3F. In other words, exosomes from unexposed MSCs appeared to have smoother surfaces, whereas TME-exposed exosomes displayed increased surface heterogeneity.

### Nanomechanical Characterization of MSC-Derived Exosomes Under TME and Non-TME Conditions

**Figure 4:**
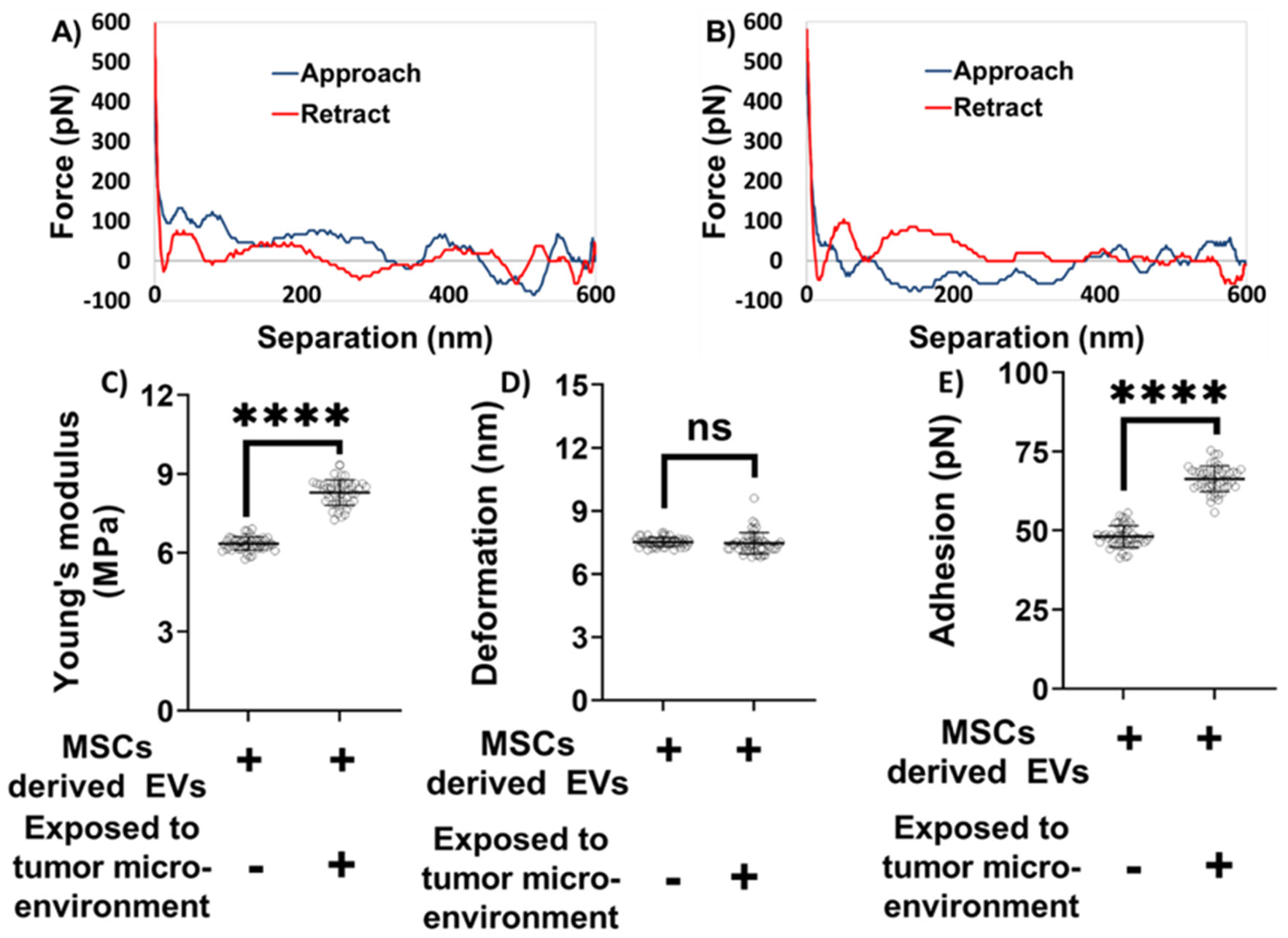
Nanomechanical characterization of mesenchymal stem cell (MSC) derived exosomes under various treatments. **A representative force-separation curve for exosomes derived from MSCs and** A) Not exposed to tumor microenvironment. B) Exposed to tumor microenvironment. **Nanomechanical attributes** C) Young’s modulus. D) Deformation. E) Adhesion. (Statistical significance performed by One Way ANNOVA: ns, not significant; ****, p<0.0001).

We next focused on evaluating the nanomechanical properties of exosomes derived from MSCs under different conditions. Nanoindentation experiments were performed on exosomes obtained from both TME-exposed and non-exposed MSCs. In these experiments, each interaction between the AFM tip and the exosome surface generated a force–separation (F–S) curve. Representative F–S curves for non-exposed and TME-exposed MSC-derived exosomes are shown in Figures 4A and 4B, respectively. A rigorous optimization process was undertaken to determine the most informative ramping parameters, based on our previous studies [22-25]. The accuracy of nanomechanical measurements depends on two interrelated factors: (i) the quality and reliability of the F–S curve, and (ii) ensuring that the indentation depth remains within 20% of the exosome height to prevent excessive deformation [39]. A reliable F–S curve is characterized by a nearly flat baseline, representing negligible force during both the approach (trace) and retraction (retrace) phases when the tip is not in contact with the sample. This non-contact region is used to correct for baseline offsets.

Based on the indentation analysis model, trace and retrace curves are used to extract Young’s modulus in various analysis models, herein Derjaguin-Muller-Toporov (DMT) model. Deformation parameter is of prime importance and is determined based on the distance between the points corresponding to slope change in trace curve and the peak force. When the tip interacts with the exosome sample during trace curve, it experiences an attractive pull towards the exosome sample, and the tip begins indenting. Thereupon, the tip continues to indent till it reaches a peak user defined force, indicated by an increasing force in the trace curve. Following which, the tip starts experiencing retractive force exerted by the piezo, indicated by a decreasing force value corresponding to the retractive curve. Adhesion parameter is the result of the attractive pull experience by the tip as it retracts from the exosome surface and hence measured from the retrace curve. As illustrated in Figures 4A-4B, the blue line represents the approach (trace) curve, where the tip moves toward the sample, while the red line denotes the retraction (retrace) curve. Quantitative nanomechanical analysis was performed by fitting the retrace curve using the DMT model. As shown in Figure 4C, exosomes derived from MSCs not exposed to the TME were significantly softer compared to their TME-exposed counterparts. Specifically, the Young’s modulus of non-exposed exosomes was measured at 6.34 ± 0.25 MPa, whereas TME-exposed exosomes exhibited a higher modulus of 8.29 ± 0.47 MPa. No statistically significant difference in deformation was observed between the two groups. The deformation values were 7.51 ± 0.21 nm for non-exposed exosomes and 7.46 ± 0.50 nm for TME-exposed exosomes (Figure 4D). However, significant differences were noted in adhesion forces. Exosomes derived from MSCs not exposed to the TME exhibited an average adhesion force of 48.12 ± 3.38 pN, which was considerably lower than that of TME-exposed exosomes, which measured 66.36 ± 4.03 pN (Figure 4E). Overall, these distinct nanomechanical attributes—especially differences in stiffness and adhesion—highlight potential biophysical markers to distinguish exosomes derived from MSCs exposed to the TME from those that are not.

### PCA detailing exposure-effect on exosomes

**Figure 5:**
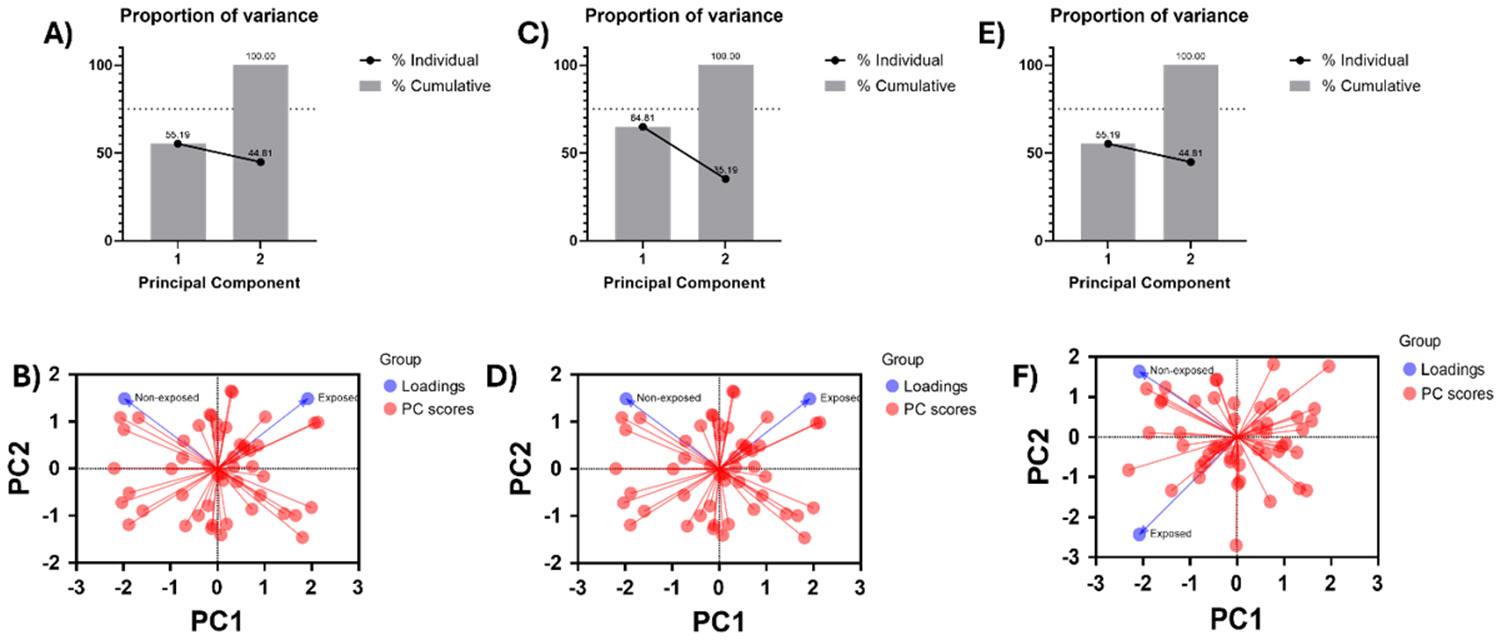
Principal Component Analysis yielding the exposure effect of condition media on nanomechanical attributes of exosomes. A) Percentage-variation and B) PC loading sourced from stiffness. C) Percentage-variation and D) PC loading sourced from deformation. E) Percentage-variation and F) PC loading sourced from adhesion.

We performed Principal Component Analysis (PCA) on nanomechanical attributes such as stiffness, deformation and adhesion to identify the effect of exposure of condition media from HTB172 lung cancer cell culture on the MSC-derived exosomes as shown in Figure 5. Nanomechanical attribute raw data was two-dimensional resulting into two principal components (PC1 and PC2) for each attribute. From stiffness data we observed 61.83 % variability in PC1, which was the largest source of variation understandably coming from the heterogeneity associated with the biological samples. PC2, orthogonal to PC1, demonstrates the biological effect of exposure of condition media and amounts to variation of 38.17% as seen from Figure 5B. Similarly, deformation yielded 64.81% and 35.19% variability in PC1 and PC2 (Figure 5C). Percentage variation in PC2 demonstrated by the PCA revealed separation between non-exposed and exposed samples primarily along PC2, also confirmed by the loading graph in Figure 5D. Lastly, adhesion did not yield a significant variation in percentage variation as seen from Figure 5E and confirmed from the loading in Figure 5F indicating the exposure effect of condition media does not have a significant effect on adhesion of exosomes. To summarize, stiffness and deformation was observed to have a detrimental effect on MSC-derived exosomes upon exposed to TME.

## Discussion

In this study, AFM was utilized to investigate morphological and nanomechanical differences between exosomes derived from MSCs cultured in the presence or absence of conditioned media from HTB172 lung cancer cells to understand the influence from TME associated factors on exosomes. Our results demonstrate that exposure to conditioned media induces significant alterations in exosomal height, surface roughness, stiffness, and adhesion, indicating that TME associated factors actively modulate the biophysical properties of MSC-derived exosomes. MSC derived exosomes are increasingly recognized as critical mediators within the TME, where they can exert either pro-tumorigenic or antitumorigenic effects depending on contextual molecular and cellular cues [40]. This functional duality positions MSC derived exosomes as both therapeutic targets and promising vehicles for drug delivery and regenerative medicine applications [41,42].

Morphological analysis revealed that exosomes exposed to conditioned media were significantly smaller, though there was insignificant difference in surface heterogeneity compared to non-exposed counterparts. Such topographical variability likely reflects changes in lipid composition, protein cargo, or membrane associated interactions, all of which are known to be influenced by environmental stressors present in the TME [43,44]. Consistent with these observations, TME exposed exosomes displayed increased stiffness and adhesion forces, suggesting that pathological conditions alter not only exosomal molecular composition but also the physical state of their membranes. AFM was central to these findings, offering distinct advantages over conventional electron microscopy techniques. Unlike methods that require dehydration or staining, AFM allows imaging in near physiological, aqueous conditions, thereby preserving native ultrastructural features and minimizing preparation related artifacts [21]. In addition to high spatial resolution, AFM enables direct measurement of nanomechanical properties, including stiffness, elasticity, and viscoelastic behavior. This capability has proven valuable in studies of diverse membrane systems such as mitochondrial cristae, lysosomes during autophagy, and the nuclear envelope under mechanical stress or disease states [45,46]. Moreover, AFM’s ability to quantify interaction forces and monitor membrane remodeling in real time provides unique insight into how exosomal membranes adapt under pathological conditions. Notably, the observed differences in nanomechanical parameters, particularly Young’s modulus and adhesion force, highlight their potential utility as biophysical biomarkers for distinguishing exosome subpopulations. Such physical signatures could be incorporated into diagnostic or therapeutic assessment workflows to identify disease associated vesicles or evaluate treatment response, especially in cancers where exosomes contribute to tumor progression, immune modulation, and metastasis [47,48].

The PCA-derived segregation of MSC derived exosomes exposed to HTB172 conditioned media can be directly interpreted in the context of AFM-based force spectroscopy measurements. The dominant contribution of stiffness (Young’s modulus) and deformation to separation along PC2 indicates that tumor-associated factors primarily alter the resistance of the exosomal lipid bilayer to AFM tip induced indentation, reflecting changes in membrane elasticity and structural integrity. Increased stiffness behavior suggests tighter lipid packing, cytoskeletal associated protein enrichment, or compositional shifts in membrane lipids such as cholesterol, all of which would manifest as higher force requirements for indentation during AFM probing. In contrast, adhesion forces measured during AFM tip retraction and reflective of nonspecific and ligand mediated surface interactions did not show significant PCA driven separation, indicating that exposure does not markedly alter surface adhesive moieties detectable under these experimental conditions, a contrasting observation from Figure 4E. Currently, it would be too primitive to comment on the contrasting behavior between Figure 4E, 5E and 5F and needs further experimental work. Together, the AFM resolved nanomechanical signatures captured by PCA demonstrate that -conditioned media from lung cancer selectively reprogram the intrinsic mechanical properties of MSC derived exosome membranes rather than their surface interaction forces, underscoring the sensitivity of AFM to detect subtle, TME induced membrane remodeling.

These findings also have important implications for the development of exosome based drug delivery platforms. Owing to their lipid bilayer structure, exosomes are well suited for transporting both hydrophobic and hydrophilic therapeutic agents [45]. Alterations in height, membrane roughness, stiffness and adhesion may directly influence drug loading efficiency, cargo retention, and cellular uptake, key determinants of therapeutic performance. Recent studies have shown that MSC derived exosomes can be engineered with surface ligands to enhance targeting specificity in diseases such as glioblastoma and Alzheimer’s disease [49-51]. AFM based biophysical characterization may therefore inform the optimization of exosome engineering and production by providing realtime assessment of membrane integrity and surface properties. Despite the growing promise of exosome-based therapies, significant challenges remain, including the standardization of exosome isolation, optimization of drug loading strategies, and reproducibility across clinical settings [46,52]. The label-free, high resolution insights provided by AFM may help address these challenges by serving as a complementary quality control tool for evaluating exosome consistency and functionality.

## Conclusion

This study demonstrates that the condition media from HTB172 lung cancer cell culture exerts a significant impact on the morphology and nanomechanical characteristics of MSC-derived exosomes. Using the high spatial and force resolution of AFM, we detected distinct changes in exosome height, surface roughness, stiffness, and adhesion under tumor associated conditions. These biophysical modifications reflect adaptive responses of exosomes to the tumor milieu and highlight their potential utility as diagnostic biomarkers and therapeutic vectors. Additionally, our findings emphasize the versatility of AFM for probing membrane bound nanostructures and support its expanding role in exosome engineering and targeted drug delivery strategies.

## Supplementary Material

1
