# Supplementary material for: An Atomic Force Microscopy analysis of Exosomes derived from Tumor Associated pluripotent Mesenchymal Stem Cells
Source: Fortune J Health Sci. Author manuscript; Available in PMC 2026 Feb 10. (PMC12885364; doi:10.26502/fjhs.388)
Supplement: 1 [file NIHMS2137372-supplement-1.pdf]

Supplementary Figure

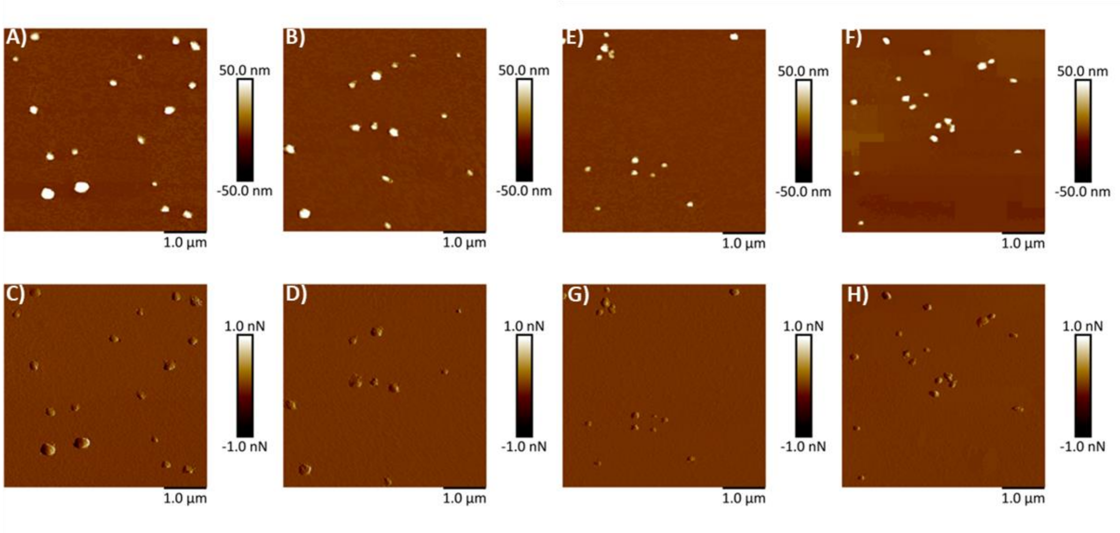

Figure S1: Representative surface topography images of mesenchymal stem cells derived exosomes under various conditions. A, B) Height image and C, D) Peak force error image of **exosomes non-exposed to tumor microenvironment (TME)**. E, F) Height image and G, H) Peak force error image of **exosomes exposed to TME.**
